# Sex in the wild: repeated observations of planktonic ciliate conjugation from field samples

**DOI:** 10.1093/plankt/fbac012

**Published:** 2022-02-28

**Authors:** Filomena Romano, Katerina Symiakaki, Paraskevi Pitta

**Affiliations:** Institute of Oceanography, Hellenic Centre for Marine Research, PO Box 2214, 71003 Heraklion, Greece; University of Copenhagen, Marine Biological Laboratory, Strandpromenaden 5, Helsingør DK-3000, Denmark; Institute of Oceanography, Hellenic Centre for Marine Research, PO Box 2214, 71003 Heraklion, Greece; Leibniz Institute of Freshwater Ecology and Inland Fisheries (IGB) Dep. 3, Plankton and Microbial Ecology Zur alten Fischerhütte 2 16775 Stechlin/OT Neuglobsow, Germany; Institute of Oceanography, Hellenic Centre for Marine Research, PO Box 2214, 71003 Heraklion, Greece

**Keywords:** ciliate conjugation, planktonic ciliates, field samples, synchronized life cycle

## Abstract

Ciliate conjugation is considered a rare event to encounter in the field and it is mostly reported from cultures. In this work, we describe a synchronized conjugation event of planktonic ciliates that was discovered twice; in September 2019, at two different locations in the Cretan Sea, Eastern Mediterranean, and in October 2020. In 2019, first, at 2 m depth of the coastal station POSEIDON-HCB, in samples fixed with acid Lugol and formaldehyde, we found 340 and 200 mating pairs L^−1^of different ciliate species, respectively; and second, at the Heraklion port, we found 220 mating pairs L^−1^ of *Strombidinopsis* sp. and 1960 mating pairs L^−1^ of *Strombidium* sp. At the Heraklion port visited again in 2020, we found 800 mating pairs L^−1^ of *Strombidinopsis* sp. and 200 mating pairs L^−1^ of *Strombidium* sp. Since detailed descriptions of conjugation in pelagic oligotrich ciliates are missing, our observations indicate that ciliate conjugation could be a frequent and periodic phenomenon, under specific conditions.

## INTRODUCTION

Ciliates are single-celled eukaryotic microorganisms that are very important for marine systems and the microbial food web (Lynn and Montagnes, 1991; Pierce and Turner, 1992). Their sexual reproduction phase is characterized by conjugation (Dini and Nyberg, 1993; Miyake, 1996; Komori *et al.*, 2002), during which two cells unite in a mating pair and fuse their cell membranes to form a cytoplasmatic bridge, a process needed for the exchange of genetic material (Miyake, 1996; Komori *et al.*, 2002). Conjugation is controlled by the mating type system (Lucchesi and Santangelo, 2004), under which two cells can conjugate only if they are part of a nonidentical mating type. Through this process, there is a change in the genetic diversity of the population because new genetic combinations are created.

Most of the studies on ciliate conjugation are conducted in cultures with widely studied genera being *Paramecium*, *Tetrahymena*, *Chilodonella*, *Oxytricha*, and *Euplotes* (Kay, 1946; Barnett, 1966; Dini and Luporini, 1979; Ricci, 1981; Bruns, 1986; Berger and Rahemtullah, 1990). In contrast, little is known for the life cycle and especially the sexual reproduction of oligotrichs, which are an important part of pelagic ciliates. In the field, ciliate conjugation has only been found in benthic ciliate communities in the Mediterranean Sea (Lucchesi and Santangelo, 2004).

In this work, we describe a synchronized conjugation event of pelagic ciliates, discovered by chance, that took place at two different locations in the Cretan Sea (Eastern Mediterranean), during September 2019. Subsequently, we found another conjugation event in October 2020.

## METHODS

On 25 September 2019, we collected water with a bucket from the port of Heraklion (35.342^o^N, 25.137°E) for educational purposes. Upon the observation of a synchronized conjugation event of oligotrich ciliates, a 50 mL sample was fixed with acid Lugol at 2% v/v final concentration. Furthermore, we isolated a live *Strombidinopsis* sp. mating pair on a petri dish for further examination, and recorded it under a Zeiss stereoscope at 40× magnification. The following day, water from the port was collected again.

Independently, on 29 September 2019, we collected two series of microplankton samples from several depths using Niskin bottles, as part of a monthly monitoring program at the coastal station POSEIDON-HCB (35.426°N, 25.072°E). The two series of samples were fixed with 2% acid Lugol and 2% formaldehyde (v/v final concentration), respectively. All samples were analyzed through an inverted microscope at 150× magnification.

In September 2020, we revisited the port of Heraklion for a second survey. Live samples were collected with a bucket, and observed daily from the 21 September, until a conjugation event was found on the 1 October. The conjugation sample was fixed with 2% acid Lugol. All the environmental variables were measured with a CTD.

## RESULTS

In both stations, temperature and salinity fell in the same ranges, between 25.35°C and 26.15°C, and between 39.74 PSU and 39.46 PSU, respectively for 2019 and 2020 in the port of Heraklion. Temperature and salinity in HCB ranged between 25.16°C and 16.66°C and between 39.38 and 39.20 PSU, respectively. Chlorophyll instead ranged between 0.02 mg m^−3^ and 0.14 mg m^−3^ in HCB and between 0.10 mg m^−3^ and 0.11 mg m^−3^, respectively for 2019 and 2020 in the port of Heraklion, Table I).

**Table I:** Environmental data of POSEIDON-HCB coastal station in 2019 and the port of Heraklion in 2019–2020

Year	Station	Depth (m)	Temperature ^o^C	Salinity PSU	Chlorophill mg m^−3^
2019	HCB	2	25.16	39.38	0.02
2019	HCB	10	25.11	39.38	0.04
2019	HCB	20	25.10	39.37	0.07
2019	HCB	50	20.17	39.00	0.20
2019	HCB	75	18.04	39.12	0.24
2019	HCB	100	17.02	39.23	0.24
2019	HCB	120	16.66	39.20	0.14
2019	Port of Heraklion	Surface	25.35	39.74	0.10
2020	Port of Heraklion	Surface	26.15	39.46	0.11

**Fig. 1:**
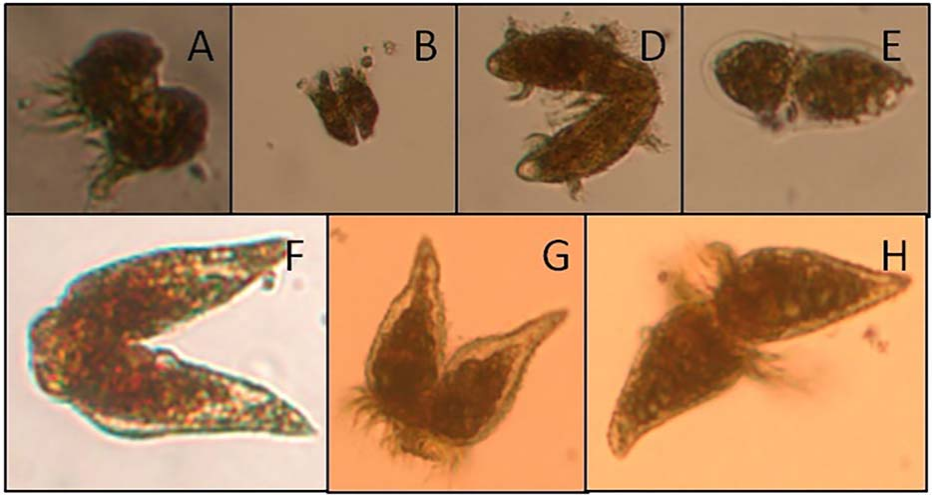
Photos of ciliates conjugating during September 2019 at POSEIDON-HCB (A, B, D), September 2019 at Heraklion port (E, F) and September 2020 at Heraklion port (G, H). *Strombidium* sp*.* (A, B), *Didinium* sp*.* (D), *Ascampbeliella* sp. (E), *Strombidinopsis* sp*.* (F, G, H).

In the 2019 Heraklion port sample, we found 220 mating pairs L^−1^ of *Strombidinopsis* sp*.* (Fig. 1, Table II) and 1960 mating pairs L^−1^ of *Strombidium* sp. In contrast, ciliates were completely absent in the sample of the following day.

**Table II:** Number of mating pairs L^−1^ for different ciliate species in the Lugol and formaldehyde fixed samples from the POSEIDON-HCB station

Station-Year	Species	Lugol Mating pairs L^−1^	Formaldehyde Mating pairs L^−1^
HCB-2019	*Strombidinopsis* sp*.*	10	
HCB-2019	*Strombidium* sp*.*	290	60
HCB-2019	*Didinium* sp*.*	10	10
HCB-2019	*Eutintinnus* sp*.*	20	30
HCB-2019	*Ascampbeliella armilla*	10	
HCB-2019	Other		100
Port of Heraklion-2019	*Strombidinopsis* sp.	220	
Port of Heraklion-2019	*Strombidium* sp.	1960	
Port of Heraklion-2020	*Strombidinopsis* sp.	800	
Port of Heraklion-2020	*Strombidium* sp.	200	

During most of the observation period, the two cells of the isolated *Strombidinopsis* mating pair (Supplementary video 1) were attached to each other, and intracellular changes were noticeable on at least one of the cells. After 5 hours, one of the ciliates detached itself and swam away, whereas the other stayed on the bottom of the petri dish. The remaining cell had an altered form and continued to show changes in the arrangement of organelles and ultimately, died possibly due to increased water temperature in the petri dish after many hours over the lamp.

From the POSEIDON-HCB, conjugating ciliates were found only in the 2 m depth sample, which had the highest abundance of the entire profile (Romano and Pitta, 2021). We found 340 mating pairs L^−1^ (19% of total ciliate abundance) of different ciliate species in the Lugol, and 200 mating pairs L^−1^ (13% of the total number) in the formaldehyde sample (Table II, Fig. 1). The reduced number of conjugants in the formaldehyde sample may be explained by the reduction effect of this fixative on the ciliate abundance (Karayanni *et al.*, 2004; Romano *et al.*, 2021).

In the sample of October 2020 from the port of Heraklion, we found 800 mating pairs L^−1^ of *Strombidinopsis* sp*.* (Fig. 1) and 200 mating pairs L^−1^ of *Strombidium* sp*.* No conjugation event was found in the previous days but just few ciliate speciments.

## DISCUSSION

Detailed description of conjugation in oligotrich ciliates is missing. The phenomenon was observed for two consecutive years at the same period, indicating periodicity. Studies in cultures have reported a rhythmic occurrence of the phenomenon (Barnett, 1966; Miyake and Nobili, 1974) and Lucchesi and Santangelo (2004) suggested it could be a frequent event in natural environments. Our observations showed that ciliate conjugation is not as rare as previously thought, and indeed it could be periodic.

We found synchronized conjugation on more than one ciliate species, indicating that these cells were all at the same stage of their cell cycle. Since this phenomenon was repeatedly observed in the field, it is almost impossible that all specimens were synchronized by chance. Instead, we hypothesize that different ciliate populations respond to the same environmental stimuli and consequently “communicate” to regulate their cell cycle and undergo sexual reproduction together.

In both stations, temperature and salinity were high and this environmental status could affect the cell cycle of ciliates. On the other hand, chlorophyll a was very low at 2 m depth in HCB and higher at the surface layer of the port of Heraklion. Most probably the interaction between different environmental factors allowed ciliates of different species to undergo sexual reproduction. Moreover, during our observations, conjugation occurred in the late summer after the ciliate bloom (Romano *et al.*, 2021; Romano and Pitta, 2021), so it is possible that the population grew until a certain point and then conjugation started before the abundance dropped. Synchronization of conjugation between cells of the same species is a frequently described phenomenon in ciliate cultures (Woodard *et al.*, 1966; Wolfe, 1974; Doerder and DeBault, 1975; Dini and Luporini, 1979).

Based on our observation, ciliate conjugation occurred only at the surface layer of the water column. Since conjugation happens at the end of the cell cycle (Dini and Nyberg, 1993), it is possible that cells had stored specific organic compounds which allowed them to control their buoyancy and stabilize themselves during conjugation at the very surface.

## CONCLUSION

Our findings demonstrate that ciliate conjugation is not as rare as previously thought, and it may happen periodically under specific conditions (e.g. maximum population density). The events we observed were synchronized between different ciliate species and all mating pairs were located in the surface layer of the water column, possibly due to buoyant organic compounds they have stored.

## Supplementary Material

Conjugation_video_fbac012

Cil_conjugation_1_fbac012

Cil_conjugation_3_fbac012

Cil_conjugation_4_fbac012
