# Supplementary figures and images for: Sex in the wild: repeated observations of planktonic ciliate conjugation from field samples
Source: J Plankton Res. 2022 Feb 28;44(2):333–6. doi: 10.1093/plankt/fbac012 (PMC9832694; doi:10.1093/plankt/fbac012)

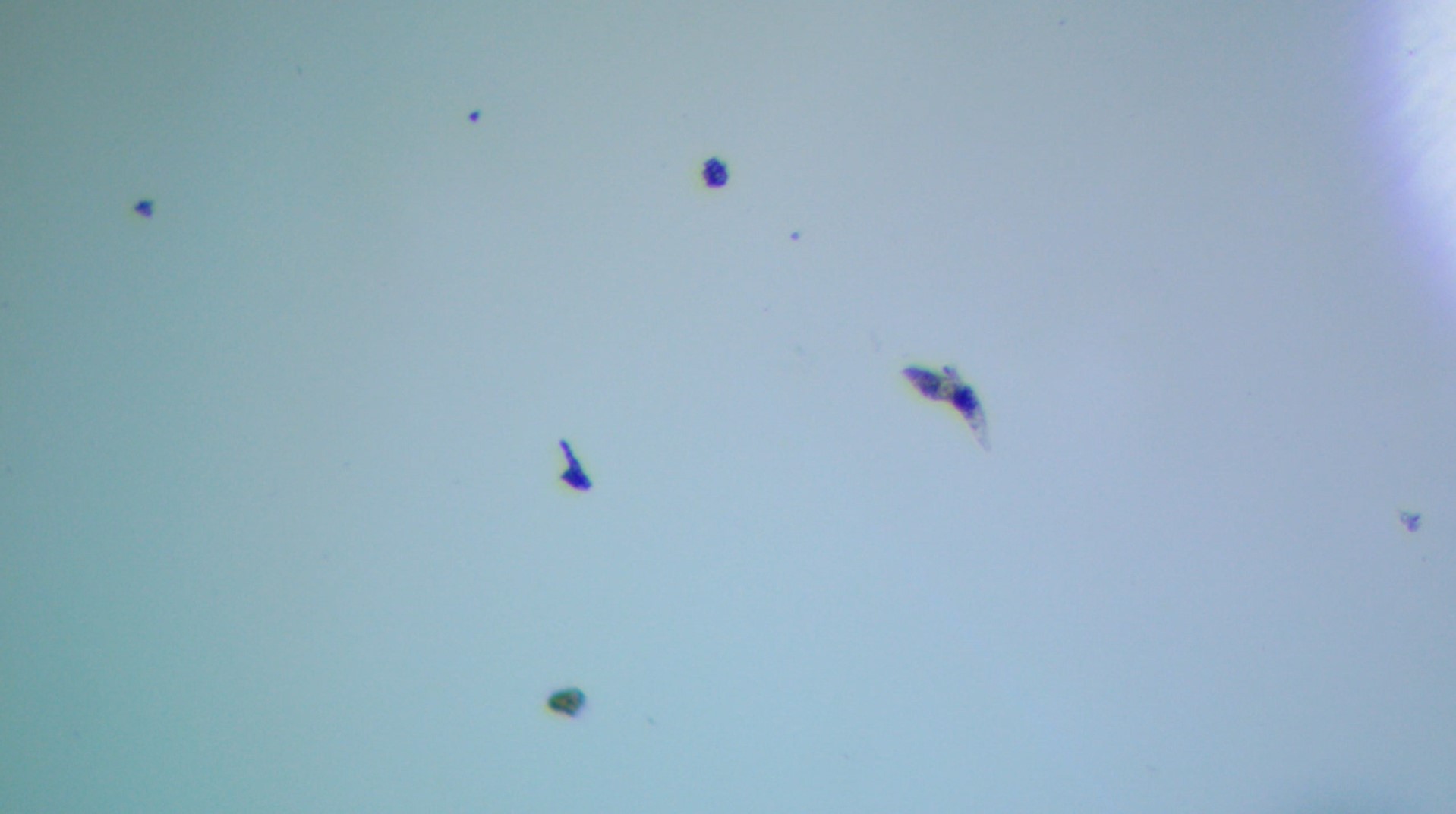

Supplement: Conjugation_video_fbac012 [file conjugation_video_fbac012.jpeg]
